# Mutational spectrum of the *SPG4 *(*SPAST*) and *SPG3A *(*ATL1*) genes in Spanish patients with hereditary spastic paraplegia

**DOI:** 10.1186/1471-2377-10-89

**Published:** 2010-10-08

**Authors:** Victoria Álvarez, Elena Sánchez-Ferrero, Christian Beetz, Marta Díaz, Belén Alonso, Ana I Corao, Josep Gámez, Jesús Esteban, Juan F Gonzalo, Samuel I Pascual-Pascual, Adolfo López de Munain, Germán Moris, Renne Ribacoba, Celedonio Márquez, Jordi Rosell, Rosario Marín, Maria J García-Barcina, Emilia del Castillo, Carmen Benito, Eliecer Coto

**Affiliations:** 1 Laboratory of Molecular Genetics -Genetic Unit, Hospital Universitario Central de Asturias, Oviedo, Spain; 2 Institute for Clinical Chemistry and Laboratory Medicine, University Hospital Jena, Jena, Germany; 3 Neurology Department, Hospital Universitari Vall d'Hebron. Univ. Autonoma Barcelona, Spain; 4 Neurology Department, Hospital 12 de Octubre, Madrid, Spain; 5 Pediatric Neurology Department, University Hospital La Paz, Madrid, Spain; 6 Neurology Department, Hospital Donostia-Instituto Biodonostia-Ciberned, San Sebastián, Spain; 7 Neurology Department, Hospital San Agustín, Aviles, Spain; 8 Neurology Department, Hospital Alvarez-Buylla, Mieres, Spain; 9 Neurology Department, Hospital Universitario Virgen del Rocio, Sevilla, Spain; 10 Department of Genetics, Hospital Universitari Son Dureta, Palma de Mallorca, Spain; 11 HGenetics Unit, Hospital Universitario Puerta del Mar, Cádiz, Spain; 12 Genetics Department, Hospital de Basurto, Bilbao, Spain; 13 Genetics Unit, Hospital Universitario Carlos Haya, Málaga, Spain

## Abstract

**Background:**

Hereditary Spastic Paraplegias (HSP) are characterized by progressive spasticity and weakness of the lower limbs. At least 45 loci have been identified in families with autosomal dominant (AD), autosomal recessive (AR), or X-linked hereditary patterns. Mutations in the *SPAST *(*SPG4*) and *ATL1 *(*SPG3A*) genes would account for about 50% of the ADHSP cases.

**Methods:**

We defined the *SPAST *and *ATL1 *mutational spectrum in a total of 370 unrelated HSP index cases from Spain (83% with a pure phenotype).

**Results:**

We found 50 *SPAST *mutations (including two large deletions) in 54 patients and 7 *ATL1 *mutations in 11 patients. A total of 33 of the *SPAST *and 3 of the *ATL1 *were new mutations. A total of 141 (31%) were familial cases, and we found a higher frequency of mutation carriers among these compared to apparently sporadic cases (38% vs. 5%). Five of the *SPAST *mutations were predicted to affect the pre-mRNA splicing, and in 4 of them we demonstrated this effect at the cDNA level. In addition to large deletions, splicing, frameshifting, and missense mutations, we also found a nucleotide change in the stop codon that would result in a larger ORF.

**Conclusions:**

In a large cohort of Spanish patients with spastic paraplegia, *SPAST *and *ATL1 *mutations were found in 15% of the cases. These mutations were more frequent in familial cases (compared to sporadic), and were associated with heterogeneous clinical manifestations.

## Background

The hereditary spastic paraplegias (HSP) are characterized by progressive spasticity and weakness of the lower limbs due to axonal degeneration in the pyramidal tract. The disease is classified as "pure" when spasticity is the only clinical finding, and as "complicated" when other clinical features (dementia, cerebellar ataxia, epilepsy, peripheral neuropathy) are also present [1]. HSP is frequently familial and at least 45 loci have been identified in families with autosomal dominant (AD), autosomal recessive (AR), or X-linked inheritance [2-4]. This genetic heterogeneity partly explains the differences in disease severity, age at onset, rate of progression, and degree of disability between families. However, intrafamilial heterogeneity is also frequent.

SPG4 (OMIM#604277) is the most common form of HSP, accounting for approximately 40% of the familial and 6-15% of the sporadic cases. The *SPAST*/*SPG4 *gene encodes spastin, a member of the AAA (ATPase associated with various cellular activities) family of proteins, implicated in the remodeling of protein complexes upon ATP hydrolysis and the coordination of axonal microtubule interactions with the tubular endoplasmic reticulum network [5,6]. To date, >240 *SPAST *mutations have been reported (http://www.hgmd.cf.ac.uk/ac/index.php; date of consultation August 2, 2010), mainly in patients with pure HSP [7-12]. Most of the *SPAST *mutations are single-nucleotide changes or small deletions/insertions, but large deletions and duplications have also been reported [13-16]. This suggested that both, haploinsufficiency and "toxic" gain of function could explain the pathogenic mechanism of *SPAST *mutations [5,17-19].

SPG3A (OMIM#606439) is the second most frequent form of ADHSP. The *ATL1/SPG3A *gene encodes atlastin, a protein localized in the endoplasmic reticulum and the Golgi and implicated in vesicle trafficking and neurite outgrowth [20,21]. To date, >25 *ATL1 *mutations have been reported (http://www.hgmd.cf.ac.uk/ac/index.php; date of consultation August 2, 2010), mainly missense changes that supported a gain of function pathogenic mechanism. *ATL1 *mutations accounted for approximately 10% of the ADHSP families, and have been mainly found in pure HSP [22,23]. *ATL1 *mutations are also frequent in early onset (childhood or adolescence) cases [24-26].

This is the first report of the mutational spectrum of the *SPAST *and *ATL1 *genes in a large cohort of unrelated HSP patients from Spain. Few studies on cohorts >200 patients have been published, and the parallel screening of both genes was rarely reported.

## Methods

### Patients

This study was approved by the Ethical Committee of Hospital Universitario Central Asturias (HUCA), and all the participants (patients and controls) signed an informed consent. A total of 370 non-related patients (index cases) were recruited through the Neurology Departments of several Hospitals from Spain. HSP was diagnosed by qualified neurologists on the basis of Harding's criteria. Based on the clinical, radiological, and biochemical findings, cases with diseases that mimicked spastic paraplegia were excluded [27]. A total of 141 patients (38%) had a family history of HSP with a dominant inheritance pattern, and were thus classified as ADHSP cases. The absence of family history of the disease was established in 229 patients (62%) after interview on their first and second degree relatives. These cases were classified as "apparently" sporadic or with an uncertain inheritance pattern. In 177 of these the two parents were alive and did not have symptoms consistent with HSP, while in 52 the inheritance pattern could not be established because no clinical data were available from relatives.

### *SPAST *and *ATL1 *sequencing

DNA was obtained from blood leukocytes and the 17 coding exons of *SPAST *(plus at least 50 bp of the flanking intronic sequences) were polymerase chain reaction (PCR) amplified (Additional file 1, Table S1). PCR fragments were purified and the two strands were sequenced using Big Dye chemistry in an ABI3130 system (Applied Biosystems, Ca, USA). These sequences were compared with the *SPAST *reference sequence (ENSG00000021574 for the genomic; ENST00000315285 for the transcript; http://www.ensembl.org). In patients who were negative for *SPAST *mutations and had ADHSP (n = 88) or patients without a family history of HSP and onset age ≤20 years (n = 99), we amplified and sequenced the 13 coding exons of *ATL1 *(reference sequences ENSG00000198513 for the genomic, and ENST00000358385 for the transcript; http://www.ensembl.org). All the DNA sequence variants were named following the guidelines of the Human Genome Variation Society (http://www.hgvs.org/mutnomen).

### Controls screening

All the new putative mutations (not previously reported as *SPAST *or *ATL1 *mutations or polymorphisms) were screened in 400 controls. These were healthy individuals aged 21-65 years recruited through the Blood Bank of HUCA. The new nucleotide changes were genotyped through PCR-RFLP, single strand conformation analysis (SSCA), or denaturing high performance liquid chromatography (DHPLC), as reported [28]. Each PCR fragment containing a putative mutation gave a characteristic RFLP, SSCA or DHPLC pattern, and we could thus determine its presence/absence in the controls. When a new nucleotide change was not found among the 400 controls we determined, when it was possible, the carrier status of all the available affected relatives to confirm the segregation of the mutation with the disease.

### MLPA analysis

The multiplex ligation dependent probe amplification (MLPA) assay with the Salsa Kit P165 HPA (MRC Holland, Amsterdam) was used to determine the existence of genome copy number aberrations in the *SPAST *and *ATL1 *genes in ADHSP patients who were negative for sequencing mutations [13]. To investigate the consequences of large *SPAST *deletions at the transcript level, we isolated the mRNA from leukocytes in 10 ml of blood (Trizol reagent, Invitrogen, Carlsbad, CA, USA) and synthesised the cDNA (Quantitec Reverse Transcripcion kit, Quiagen, Hilden, Germany). The cDNA was amplified with primers that matched exons flanking the deletion (conditions available upon request), and the PCR products were purified and sequenced.

### Analysis of *SPAST *transcripts

To determine the effect of some *SPAST *mutations on mRNA transcripts, the cDNA synthesised from leukocytes was amplified with primers that matched the exons flanking the mutation (additional file 1, Table S2), and the PCR fragments were purified from agarose gels and sequenced.

### Statistical analysis

The analysis of variance (SPSS 17.0 software) was used to compare the mean onset age and disease duration between patients with mutations in *SPAST *and *ATL1 *and patients without mutations.

## Results

### Patients characteristics

We studied a total of 370 unrelated HSP index cases, with a mean onset age of 28 (± 21) years (range 1-77 years), and a mean disease duration of 17 (± 15) years. A total of 321 patients (87%) had a pure HSP, while 49 (13%) were complicated cases with peripheral neuropathy (the most frequent finding), cerebellar or cerebral atrophy, mental retardation, nystagmus, or dysarthia.

A total of 44 of the 141 patients (31%) with ADHSP had a *SPAST *mutation, and 10 mutations were found among the 229 (5%) cases apparently sporadic or with an uncertain inheritance pattern. However, in three of these patients the mutation (p.Leu426Val, p.Lys503insArg, and p.Met390Val) was *de novo *because none of the two parents was mutation carrier (the paternity was confirmed). We found 10 *ATL1 *mutation carriers among 88 ADHSP cases (11%), and the only apparently sporadic proband had a *de novo *mutation (p.Gln154Glu) (Additional file 2, Figure S1).

### *SPAST *mutations

**Figure 1:**
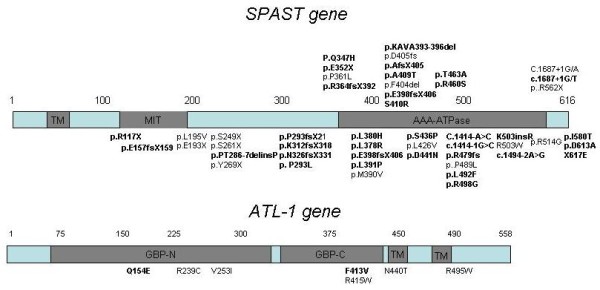
**Schematic diagram of *SPAST *and *ATL1 *domains showing the location of the mutations**. The one letter code was used to identify the amino acid changes. Novel mutations are in bold.

We found 48 *SPAST *mutations in 52 patients (Table 1). These were missense changes in amino acids conserved between species (n = 22), nonsense mutations (n = 7), small frameshifting insertions/deletions (n = 13), nucleotide changes in the intronic splicing consensus sequence (n = 5), and a sense mutation in the stop codon (Figure 1). A total of 15 mutations had been previously reported, while 33 (69%) were novel.

**Table 1:** Mutations identied in the *SPAST *gene in the Spanish HSP cohort.

Case	Onset age (Years)	Phenotype*	GENE	EXÓN	Mutation	Protein change	Reference
193	60	Pure-UHSP	*SPAST*	1	c.349C->T	p.Arg117X	This study
69	45	Pure-SHSP	*SPAST*	2	c.469delG	p.Glu157f sX159	This study
81	35	Pure-SHSP	*SPAST*	3	c.577C>T	p.Gln193X	[7]
138	2	Complicated-ADHSP	*SPAST*	3	c.583C->G	p.Leu195Val	[10]
166	43	Pure-ADHSP	*SPAST*	5	C.782C>G	p.Ser261X	[7]
197	53	Pure-ADHSP	*SPAST*	5	c.857-859delCTA	p.Pro286-Thr287delinsP	This study
257	23	Pure-ADHSP	*SPAST*	5	c.806c->G	Tyr269X	[7]
300	8	Pure-ADHSP	*SPAST*	5	c.746C>G	p.Ser249X	[8]
							
174	1	Pure-ADHSP	*SPAST*	6	c.879delG	p. Pro293fsX314	This study
205	20	Pure-ADHSP	*SPAST*	6	.936-37insA	pLys.312fsX318	This study
206	30	Pure- UHSP	*SPAST*	6	c.977-978insA	p.Asn326fsX331	This study
							
335	32	Pure- SHSP	*SPAST*	6	c.878C>T	p.Pro293Leu	This study
13	40	Pure-ADHSP	*SPAST*	7	c.1040A>C	p.Gln347His	This study
130	0	Complicated-ADHSP	*SPAST*	7	c.1082C>T	p.Pro361Leu	[41]
133	35	Pure-ADHSP	*SPAST*	7	c.1054C>T	p.Gln352X	This study
351		Pure-ADHSP	*SPAST*	7	c. 1091-1098delGGCCTGAG	p.Arg364fsX392	This study
47	66	Pure-ADHSP	*SPAST*	8	c.1139T>A	p.Leu380His	[15]
111	49	Pure-ADHSP	*SPAST*	8	c.1133T->G	p.Leu378Arg	This study
180	54	Pure-ADHSP	*SPAST*	8	c.1172T>C	p.Leu391Pro	[42]
273	1	Complicated-SHSP	*SPAST*	8	c.1168A->G	p.Met390Val	[43]
7	44	Pure-ADHSP	*SPAST*	9	c.1215-1219delTATAA	p. Asn405fsX441	[7]
22	42	Pure-ADHSP	*SPAST*	9	c.1174-1180delGCTAAG	p.Ala392fsX405	This study
23	35	Pure-ADHSP	*SPAST*	9	c.1226G>A	p.Ala409Thr	This study
32	35	Pure-ADHSP	*SPAST*	9	c.1177-1180delAAAGCAGTA GCT	p.Lys393-Ala396del	This study
52	45	Pure-ADHSP	*SPAST*	9	c.1210-1212delTTT	p.Phe404del	[44]
80	38	Pure-ADHSP	*SPAST*	9	c.1210-1212delTTT	p.Phe404del	[44]
98	29	Pure-ADHSP	*SPAST*	9	c.1192-93delGA/insT	Glu398fsX406	This study
179	10	Pure-ADHSP	*SPAST*	9	c.1192-93delGA/insT	Glu398fsX406	This study
363	2	Pure_ADHSP	*SPAST*	9	C.1128A>C	p.Ser410Arg	This study
135	47	Pure-ADHSP	*SPAST*	10	c.1306T>C	p.Ser436Pro	This study
170	3	Complicated-SHSP	*SPAST*	10	c.1276 C>G	p.Leu426Val	[7]
373	10	Pure-ADHSP	*SPAST*	10	c. 1321G->A	p.Asp441Asn	This study
50	2	Pure-ADHSP	*SPAST*	11	c.1387A>G	p.Thr463Ala	This study
200	35	Pure-ADHSP	*SPAST*	11	c.1378c>A	p.Arg460Ser	[45]
308	30	Pure -ADHSP	*SPAST*	IVS11	c.1414-2A>C	Exon 12 skipping	This study
322	30	Pure_ADHSP	*SPAST*	IVS11	c.1414-1G>C	Exon 12 skipping	This study
86	40	Pure- ADHSP	*SPAST*	12	c.1439-1145delTACTTGT/insC	p.Val480fs	This study
118	61	Pure-ADHSP	*SPAST*	12	c.1466C-> T	p.Pro489Leu	[46]
177	20	Pure-ADHSP	*SPAST*	12	c.1492A<G	p.Arg498Gly	This study
352		Pure-ADHSP	*SPAST*	12	c.1474C>T	p.Leu492Phe	This study
145	16	Pure-ADHSP	*SPAST*	IVS12	c1494-2A>G	presumed missplicing	This study
178	1	Pure-SHSP	*SPAST*	13	c.1507-1508insGGC	p.Lys503insArg	This study
334	32	Pure-ADHSP	*SPAST*	13	c.1540A>G	p.Arg503Trp	[11]
358	30	Pure-ADHSP	*SPAST*	14	c.1540A>G	p.Arg514Gly	[12]
253	18	Pure-ADHSP	*SPAST*	15	c.1684C>T	p.Arg562X	[7]
261	50	Pure-ADHSP	*SPAST*	IVS15	c.1687+1G/A	Ex15 skipping	[7]
62	40	Pure-ADHSP	*SPAST*	IVS15	c.1687+1G/T	Ex15 skipping	This study
103	40	Pure-ADHSP	*SPAST*	17	c.1793T-> C	p.Ile580Thr	This study
112	56	Pure-ADHSP	*SPAST*	17	C.1739T-> C	p.Ile580Thr	This study
212	35	Pure-ADHSP	*SPAST*	17	c.1849T-> G	p.X617Glu	This study
286	54	Complicated-UHSP	*SPAST*	17	c.1849T-> G	p.X617Glu	This study
310	36	Pure- UHSP	*SPAST*	17	C.1838A-> C	p.Asp613Ala	This study
**EXON DELETIONS**							
199	20	Pure-ADHSP	*SPAST*	-		EX10-16 deletion	[14]
225	14	Pure-ADHSP	*SPAST*	-		Ex 6-7 deletion	This study

* ADHSP- Autosomal dominant spastic paraplegia; SHSP: apparently sporadic spastic paraplegia (including siblings with healthy parents); UHSP: Unkown familial history spastic paraplegia

### *SPAST *missense mutations out of the AAA domain

Most of the missense mutations were in the AAA domain. Two novel missense changes (p. Pro293Leu, and p. Asp613Ala) were outside of the AAA domain, where missense mutations have been rarely found. The two amino acids were evolutionary conserved. The p.Pro293Leu proband was a 51-year-old man with progressive gait disorder starting at the age of 31, who has been confined to a wheelchair due to his spasticity (pure form) for the last two years. His 46-year-old sister, and one of his daughters (18 years old) also carried this mutation. They both presented brisk reflexes and Babinski's sign, but no spasticity and an almost normal gait. The p.Asp613Ala was found in a 45 years old man with a pure phenotype affecting only the lower limbs and the first symptoms at the age of 36 years. He did not report a family history of the disease, although no relatives were studied.

We also found a new *SPAST *missense variant (p.Ile328Leu) in a 15 years old male with complicated spastic paraplegia starting at the age of 18 months. This putative mutation was also found in his father's grandmother (asymptomatic), but the proband's father was not available for study. Although this variant was not found among the controls and involved a conserved amino acid, a bioinformatics analysis (SIFT v2. program, Sorting Intolerant From Tolerant; http://sift.jcvi.org/) indicated a non significant change in the protein function, and was thus classified as a change of uncertain pathogenic effect.

### *SPAST *c.583C>G (exon 3) affected splicing

**Figure 2:**
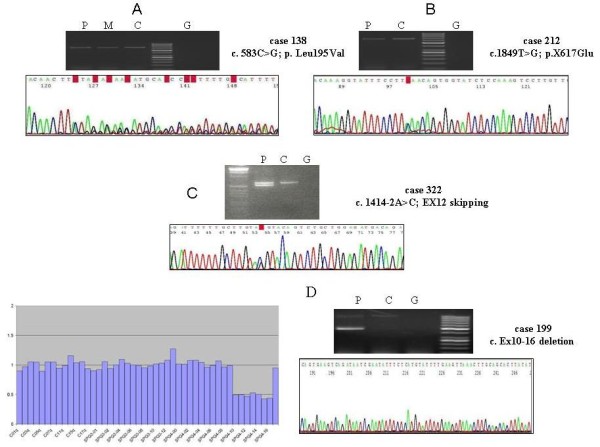
**A) The cDNA from patient 138 (carrier of c.583C>G in exon 3 of *SPAST*) was synthesised from leukocytes mRNA**. The sequencing of the fragment amplified with primers that matched exons 2 and 7 showed the deletion of the last 4 nucleotides of exon 3. **B) **The cDNA from patient 212 (X617Glu) was amplified with primers for exons 11 and 17 of *SPAST*, and the sequence showed similar electropherogram peaks for the two alleles. **C) **The cDNA of patient 322 was amplified with primers for exons 7 and 17 of *SPAST*, and the sequence showed the effect on splicing of the c.1414-1G>C mutation. **D) **MLPA of the *SPAST *gene in patient 199, showing the exons 10-16 deletion. The cDNA was amplified with primers that matched exons 9 and 17, and two PCR fragments were obtained. Sequencing of the smaller fragment confirmed the absence of exons 10 to 16. P = patient (cDNA); M = Patient's mother's (cDNA); C = Control (cDNA); G = Genomic DNA

The c.583C>G (p.Leu195Val) was found in a 19 years old male with an onset at the age of 2 years and a phenotype complicated with neuropathy. He was from a family with ADSPH in which we confirmed the segregation of the disease with this mutation. This has been previously reported as a missense mutation, and the bioinformatic analysis (SIFT v2. program) indicated that this change would affect the protein function. However, the c.583C>G was four nucleotides from the 3' end of exon 3, and was also predicted to reduce the score of the splicing consensus site (Human Splicing Finder v. 2.4; http://www.umd.be/SSF). This mutation could thus result in an aberrant mRNA sequence. To confirm this, we isolated the mRNA from the patient's blood leukocytes and amplified the *SPAST *cDNA with primers for exons 2 and 7. The last 4 nucleotides of exon 3 were missed in the transcript, that would thus be translated into an aberrant protein (Figure 2A).

### *SPAST *sense mutation

We found a novel mutation in the *SPAST *stop codon, c.1849T>G (p.X617Glu) in two non related patients without known affected relatives. The mutated transcript was predicted to be translated into a 46 amino acids longer protein. To determine the stability of the mRNA containing this mutation, we synthesized the cDNA from RNA obtained from leukocytes of one of the patients and amplified and sequenced a PCR-fragment generated with primers that matched exons 11 and the 3' untranslated region (UTR). The two alleles gave equally intense sequence electropherogram peaks, suggesting that c.1849G did not increase the mRNA decay and was likely translated into a mutated protein (Figure 2B).

### *SPAST *mutations affecting splicing

In addition to the above described c.583C>G in exon 3 of *SPAST*, we found five changes in intron splicing consensus nucleotides. The *in silico *analysis (Splice Site Prediction by Neural Network; http://www.fruitfly.org/seq_tools/splice.html) indicated that the five changes would affect the pre-mRNA splicing. We could synthesize the cDNA from four of the patients, that was amplified with primers that matched exons 7 and 17, and exons 11 and 17. In all these cases two PCR-fragments were amplified, and the longer fragment corresponded to the wild type transcript while the shorter resulted from an exon skipping. In this way, we confirmed the existence of defective transcripts (Figure 2C).

### Large *SPAST *deletions

We performed MLPA analysis in the 77 ADHSP cases who were negative for *ATL1 *and *SPAST *sequencing mutations. Two patients (2.5%) showed a significantly reduced amplification signal for exons 10-16, or exons 6-7 of *SPAST*. In the patient with a putative deletion of exons 10-16 we amplified the cDNA with primers that matched exons 9 and 17, and confirmed the skipping of exons 10-16 (Figure 2D). In this pedigree, the age at onset (third decade) was similar in the three affected members who were studied, and the oldest patient (an 81-year-old man) had been confined to a wheelchair for the previous five years.

### *ATL1 *mutations

The *ATL1 *exons were sequenced in a total 88 patients with ADHSP and in 99 patients without a family history of HSP and an onset age ≤20 years. We found seven mutations and a variant with uncertain pathogenic effect in 11 patients (Table 2). Three were novel missense changes (p.His256Asp, p.Gln154Glu, and p.Phe413Val), while 5 had been reported. The p.Arg239Cys was a recurrent mutation found in 3 non related patients.

**Table 2:** Mutations in the *ATL1 *gene.

Case	onset age (Years)	Phenotype/Inheritance	GEN	Exon	Mutation	Protein	Reference
57.1	1	Pure- SHSP	*ATL1*	4	c.460 C>G	p.Gln154Glu	This study
97	8	Pure-ADHSP	*ATL1*	7	c.715C>T	p.Arg239Cys	[47]
102	4	Pure-ADHSP	*ATL1*	7	c.715C>T	p.Arg239Cys	[47]
220	3	Pure-ADHSP	*ATL1*	7	c.715C>T	p.Arg239Cys	[47]
279	5	Pure-ADHSP	*ATL1*	7	c.715C>T	p.Arg239Cys	[47]
64	10	Pure-ADHSP	*ATL1*	8	c.757 G>A	p.Val253Ile	[25]
159	5	Pure-ADHSP	*ATL1*	12	c.1483c>T	p.Arg495Trp	[25]
110	17	Pure-ADHSP	*ATL1*	12	c.1319A->C	p.Asn440Thr	[25]
232	6	Pure-ADHSP	*ATL1*	12	c.1237T->G	p.Phe413Val	This study
233	16	Complicated-ADHSP	*ATL1*	12	c.1243 C->T	p.Arg415Trp	[40]

Mutations p.Gln154Glu, (c.460 C>G), p. His256Asp (c.766C>A), and p.Phe413Val (c.1237T>G) are located in the guanilate binding protein domains. The p.Gln154Glu mutation was found in a patient with a pure phenotype and onset of symptoms in childhood. His son showed gait problems at the age of two years. The p.His256Asp patient was a 45 years old woman with the onset of symptoms at the age of 3 years and a progressive spastic paraplegia (Additional file 2, Figure S1). In her family, we identified mutation carriers who were asymptomatic at ages of 60 and 63 years, and this could thus be classified as a variant with uncertain pathogenic effect. Mutation p.Phe413Val was found in a patient with an onset age of 6 years and a complicated phenotype (neuropathy). Her mother was affected, but she was death and we could not confirm whether carried the mutation. None of the 77 ADHSP patients studied through MLPA showed evidence for large *ATL1 *deletions.

### *SPAST *and *ATL1 *polymorphisms

We found several polymorphisms in the two genes (Additional file 1, Table S3). All the new polymorphisms were intronic, or exonic silent amino acid changes with one exception: c.844 T>A in exon 5 of *SPAST *(p.Ser282Thr). This missense change affected an amino conserved between species and was not found in any of the 400 controls. Although it could be considered a putative mutation, was in a patient with the p.Ser261X mutation (located also in exon 5). Segregation analysis to determine whether the two nucleotide changes were in different chromosomes was not possible. However, the electrophoretic pattern of bands after double restriction enzyme digestion (*MnlI *+ *MboI*) of the exon 5 fragment indicated that the mutation was *in cis *with the c.844 A allele (data not shown). This suggested that this was likely a rare variant, rather than an HSP mutation. Two missense polymorphisms that have been proposed as modifiers of the clinical phenotype (p.Ser44Leu and p.Pro45Leu) were not found in our patients [29].

### Genotype-phenotype correlation

The phenotype associated with *SPAST *and *ATL1 *mutations was pure in 48 (87%) and 10 (90%) of the index patients, respectively. We analysed all the available relatives of the 63 index cases with mutations, and identified a total of 54 *SPAST *and 15 *ATL1 *mutation carriers. A total of 13 mutation carriers (10 for *SPAST *and 3 for *ATL1 *gene) were asymptomatic at the time of our analysis. The mean onset age of the disease (index cases and relatives) was 34.5 (± 17.72) years for *SPAST *mutation carriers, and 7.67 (± 5.9 years) for *ATL1 *mutation carriers (p < 0.001). A difference was also observed between *ATL1 *mutation carriers and patients without *SPAST/ATL1 *mutations (7.67 ± 5.9 vs. 27.78 ± 20.238; p < 0.001). The mean duration of symptoms at the time of examination did not differ between patients with *SPAST *(14.5 ± 11.1) and *ATL1 *(18.78 ± 15.057) mutations. No difference in disease duration was observed between *SPAST *or *ATL1 *mutation carriers and patients without mutations.

## Discussion

*SPAST *mutations (including large gene deletions) were found in 15% (54/370) of the patients, but this frequency increased to 31% (44/141) among patients with ADHSP. Most of the *SPAST *mutations were novel, and this was in agreement with previous reports that described a high rate of private mutations in this gene [30,10,15]. We found an *ATL1 *mutation in 6% of the patients studied for this gene, but this frequency could be underestimated because we did not include ADHSP patients with a *SPAST *mutation or patients with sporadic/uncertain HSP and an onset age >20 years. However, we think that the *ATL1 *mutation rate should be right because double mutated patients (*SPAST *+ *ATL1*) have not been reported, and *ATL1 *mutations were rare among non-ADHSP patients with an onset age >20 years [22,25,26].

Considering the ADHSP cases, the mutational screening of *SPAST *and *ATL1 *identified a total of 54 mutation carriers, a frequency (38%) within the range reported in other populations [10,12,31,32]. Also in agreement with previous reports, the frequency of *SPAST *and *ATL1 *mutation carriers was much lower among patients with sporadic/uncertain HSP [12]. Four of the sporadic cases had a *de novo *mutation. A high rate of *de novo *mutational events for *SPAST *and *ATL1 *has been previously described, indicating that sporadic cases should also be screened for mutations in these genes after exclusion of other major neurological causes of spasticity [15,23,33-35].

*SPAST *mutations are mostly restricted to the AAA protein domain. It is thus remarkable that three of the missense mutations in our patients were out of this domain (p.Leu195Val, p.Pro293Leu, and p.Asp613Ala). In the case of *ATL1 *all the mutations were missense changes in the GTPase and transmembrane domains of atlastin, and would disrupt the normal protein folding and oligomerization [20,21]. The *SPAST *c.C583G variant was previously reported as a missense mutation (p.Leu195Val) [10]. However, this change was within the consensus splicing sequence of exon 3 and we confirmed its effect on pre-RNA splicing. Nucleotide changes in the last nucleotides of exons could result in both, normal and splicing defective transcripts [36-38]. Although we found an aberrant transcript we could not define whether the c.583 G resulted also in normal transcripts.

In the *SPAST *gene we also found one mutation in the stop codon (c.1849T>G; X617Glu), that would result in the translation of 46 amino acid beyond the stop codon. To our knowledge, this is the first sense mutation reported in this gene. The abolishment of a stop codon and the appearance of a longer ORF has been found in other hereditary diseases, such as the British Familial Dementia caused by a sense mutation in the *ITM2B *gene [39].

We performed MLPA analysis in 78 patients with ADHSP and we found two large deletions in the *SPAST *gene, which accounts for 2.5% of patients. In a recent work Shoukier et al. reported a similar frequency for this type of mutation [12]. However, previous studies reported a much higher frequency (18-23%) [13,15]. Additional studies are thus necessary to define the frequency of large deletions/duplications in the aetiology of HSP.

Finaly, *SPAST *and *ATL1 *mutations have been associated with variable penetrance, leading to heterogeneous HSP phenotypes in terms of onset age and clinical symptoms (pure vs. complicated) [40,15]. This heterogeneity could be partly explained by different mutated genes. As discussed above, we observed a significantly higher frequency of familial dominant HSP among *ATL1 *patients, compared to *SPAST *mutation carriers, and pure HSP was also more frequent among the patients with *ATL1 *mutations. However, phenotypic variability was also common among mutation carriers from the same family.

## Conclusions

In conclusion, we reported the mutational spectrum of the *SPAST *and *ATL1 *genes in a large cohort of Spanish patients with spastic paraplegia. We found a mutation in 15% of the cases, and a frequency of mutation carriers significantly higher among ADHSP compared to sporadic cases. Thus, the genetic screening should be more relevant in patients (pure and complicated phenotypes) with a family history of the disease. However, the fact that a significant number of apparently sporadic cases had a mutation suggested that these patients should not be excluded from the genetic study. The mutational report could be of limited value to predict the phenotype associated to these mutations, as demonstrated by the heterogeneous behavior of most of the mutations.

## Competing interests

The authors declare that they have no competing interests.

## Authors' contributions

All the authors contributed to this work by recruiting the patients, obtaining the clinical and analytical information, or performing the laboratory work. VA designated the work and analyzed the results. VA, ES-F, CB, M., BA, and AIC performed all the genetic analysis. VA and EC wrote the manuscript. All authors have read and approved the submission of this manuscript.

## Note

* Manuel Amorín, Eugenia Marzo-Sola, Carlos H. Lahoz, Pia Gallano, Concepción Alonso-Cerezo, Rafael Palencia-Luaces, Eduardo Gutiérrez- Rivas, Rogelio Simón, Loreto Martorell, Eduardo López-Laso, José M. Asensi, Luis Hernández- Echebarria, Yolanda Morgado, Alonso González-Masegosa, Juan J. Garcia- Peñas, Irene Catalina-Alvarez, José L. Muñoz-Blanco, Miguel Fernández-Burriel, Juan B. Espinal, Mariano Aparicio- Blanco, Jon Infante, María Vázquez-Espinar, Elena Maside

## Pre-publication history

The pre-publication history for this paper can be accessed here:

http://www.biomedcentral.com/1471-2377/10/89/prepub

## Supplementary Material

Additional file 1 **Supplementary tables and supplementary figure 1 legendl for Alvarez et al**. This file contains information about PCR primers, polymorphisms detected and legend of supplementary figure. Click here for file

Additional file 2 **supplementary FIGURE1 for Alvarez et al**. This file contains a figure showing several examples of mutations detected. Click here for file
