# Supplementary material for: Mutational spectrum of the SPG4 (SPAST) and SPG3A (ATL1) genes in Spanish patients with hereditary spastic paraplegia
Source: BMC Neurol. 2010 Oct 8;10:89. doi: 10.1186/1471-2377-10-89 (PMC2964648; doi:10.1186/1471-2377-10-89)
Supplement: Additional file 1 — Supplementary tables and supplementary figure 1 legendl for Alvarez et al. This file contains information about PCR primers, polymorphisms detected and legend of supplementary figure. [file 1471-2377-10-89-S1.DOC]

**Table S1.** Sequence of primers to amplify the *SPAST* and *ATL1* exons, and annealing temperatures and size of the PCR-fragments.

| *SPAST.* EXON | Primer sequence  Forward (F)  Reverse (R) | Annealing  (ºC) | | Size  (bp) |
| --- | --- | --- | --- | --- |
| 1 | F- GACAGCGACAGGAAGGGAG | 59 (10%DMSO) | | 474 |
|  | R- AGAGCCACACGAAGAGGAG |  |  |  |
|  | F- GTTTGGTCGCCTTCCACCT | 61  (10% DMSO) | | 306 |
|  | R- ACCCCACCGCCTTCTTCC |  |  |  |
| 2 | F- GATTTGCAATATTTAGTGTACTC | 54 | | 225 |
|  | R- AAATAAATAGATCTGAAATCTGG |  |  |  |
| 3 | F- TTATCGTGAAACAATATTAGTTG | 54 | | 225 |
|  | R- CTATTTCTTTCTACAAATTAAAGTTT |  |  |  |
| 4 | F- TGTTCATTATCTTTTTTCTTTTT | 54 | | 220 |
|  | R- TAAGTAAGACTCTTACTTTGCATG |  |  |  |
| 5 | F- GTTCAGCTACAATTTTCTAATCAC | 54 | | 315 |
|  | R- TATGATCAACTTAAGCAGGAAT |  |  |  |
| 6+7 | F- GTAAATGTTAGGTTGTATTTTCAT | 54 | | 630 |
|  | R- TGGATTCAGTAACAGATGGT |  |  |  |
| 8 | F- CTGTTTGGGAAGATGCTACTG | 54 | | 260 |
|  | R- CTCAAGGACAAGATAAGTTTCTT |  |  |  |
| 9 | F- GGCCTCATAGCTTACATTTTTAG | 54 | | 220 |
|  | R- ATACGACAATATTGGAAACAGAG |  |  |  |
| 10 +12 | F- CTGTGTGCTAGATTTTCAACA | 54 | | 780 |
|  | R- TGTAAGATGGACACATGAGTAA |  |  |  |
| 13 | F- ATTAACTAGAGTTTTAAAAGAGTAACAA | 54 | | 265 |
|  | R- GATGGTAGTTCTTGTTTCTGC |  |  |  |
| 14 | F- ATCATTAATTCTGAAATTAGACTGA | 54 | | 220 |
|  | R- ACTCATTTCATACTTAAGATTACTTT |  |  |  |
| 15 | F- GATCATGCCATTGCACTC | 54 | | 240 |
|  | R- GGACTTCTTAAACTTCTAAGGTG |  |  |  |
| 16 | F- TGTATGTATTTTTAAGTGCCTGAC | 54 | | 125 |
|  | R- TTACAATATAGAAGACAAAGAAAAT |  |  |  |
| 17 | F- AACCACCATATACCTGTTGA | 54 | | 300 |
|  | R- GTAAGACTCTAAAAAGTCCTGTAA |  |  |  |
| *ATL1-*EXON | Primer sequence  Forward (F)  Reverse (R) | Annealing (ºC) | Size  (bp) | |
| 2 | F- AGCATAAAATGAGCATTTATAA | 54 | 410 | |
|  | R- AAAGCACTGAGGTTGGAAT |  |  |  |
| 3+4 | F- CCTTTTAACTCGAATTGGAG | 54 | 883 | |
|  | R- CTAATTATAACATGTATGTAGAAACAC |  |  |  |
| 5 | F- GTGGTAACTGATATTTTTAAAAGT | 54 | 246 | |
|  | R- CAGAATCAGAATCTGATTATGTT |  |  |  |
| 6 | F- GAAAGAAAGCCAAGAATTAAA | 54 | 241 | |
|  | R- GAGAAGGAAACATGACCCT |  |  |  |
| 7+8 | F- ACTGGTTTCCATATAAAGCA | 54 | 226 | |
|  | R- AAATGATCCAACAGAAATAATT |  |  |  |
|  |  |  |  |  |
| 8 | F- CAGAATTCCTTTGCATAATT  R- AAAATGACATTTTTATAATCAGAC | 54 | 259 | |
| 9 | F- GAGTGATGGCATTATCACTG | 54 | 347 | |
|  | R- GTACCTTTGCTCCCATATTAT |  |  |  |
| 10 | F- GGGAATTTAAGTATGAGAAAGA | 54 | 182 | |
|  | R- ATTCTGTCTAATATTGTTATTTGG |  |  |  |
| 11 | F- ATTTTGAGGACTTTGGTTTCT | 54 | 270 | |
|  | R- GTGTGTACAGTGTAACATGGAA |  |  |  |
| 12 | F- TTTTGATACAGTTGCCAATT | 54 | 550 | |
|  | R- ACATTCAAATGTGTGCTGAA |  |  |  |
| 13 | F- TGATAAAATATGTAATCTAAACTGA | 54 | 277 | |
|  | R- TCACCAAAGATTGTTCTAATCTACA |  |  |  |
| 14 | F- TTTTACATCTGTGTGTTTAATAAA | 54 | 250 | |
|  | R- AGCACTTTGAATGTTTGCA |  |  |  |

**Table S2.** Primers to amplify the *SPAST* transcript, and annealing temperatures and size of the PCR-fragment from normal sequence transcripts.

| Exons | Primer sequence  Forward (F)  Reverse (R) | Annealing  (º) | Size  (bp) |
| --- | --- | --- | --- |
| 2-7 | 2F- 5’ GCTGTGGAATGGTATAAGAA  7R- 5’ CAGAGAAGGAAGAATAACAAT | 54ºC | 660 |
| 7-17 | 7F- 5' AATTTGATGATATAGCTGGTC**G**  17R- 5' GTAAGACTCTAAAAAGTCCTGTAA | 54ºC | 898 |
| 11-17 | 11*F-5' TTTTGTGTGAAAGAAGAGAAG  17R- 5' GTAAGACTCTAAAAAGTCCTGTAA | 54ºC | 631 |

**Table S3**. *SPAST* and *ATL1* polymorphisms, or variants with unknown effect.

| Gene | Nucleotide change | Exon/intron | SNP ID. | Amino acid |
| --- | --- | --- | --- | --- |
| *SPAST*  *ATL1* | *c.992A>T  **c.766C>A | Exon 6  Exon 8 | Novel  Novel | Ile328Leu  His256Asp |
|  | | | | |
| *ATL1* | C.84A>G | Exón 2 | rs35014209 | Pro28Pro |
| *ATL1* | c.351A>G | Exon 3 | rs17850684 | Glu117Glu |
| *ATL1* | IVS3-19G>T | Intron 3 | rs1060197 |  |
| *ATL1* | c.574 -33-34del2 | Intrón 5 | rs35968976 |  |
| *ATL1* | c.630 +7G>A | Intron 6 | rs3759588 |  |
| *ATL1* | c.863-54A>G | Intron 8 | rs11157759 |  |
| *ATL1* | c.1048 -58G>A | Intron 10 | Novel |  |
| *ATL1* | c.1552 -103G>T | Intron 12 | rs61985479 |  |
| *ATL1* | c.1552 -111TTCTT In/del | Intron 12 | rs35540751 |  |
| *SPAST* | c.391C>T | Ex1 | Novel | Leu131Leu |
| *SPAST* | c.503 +53T>C | Intrón 2 | rs7561519 |  |
| *SPAST* | c. 844T>A | Exon 5 | Novel | Ser282Thr |
| *SPAST* | c.879G>A | Exon 6 | Sauter, 2000 | Pro293Pro |
| *SPAST* | c.1374T>C | Exon 11 | Novel | Ser458Ser |
| *SPAST* | c.1722C>T | Ex15 | Novel | Ala574Ala |
| *SPAST* | c.1332 -37delG | Intron 10 | Sauter, 2000 |  |
| *SPAST* | c.1413 +42 TATAin/del | Intron 11 | rs10627985 |  |
| *SPAST* | c.1494 -3delT | Intron 12 | Depienne, 2007 |  |
| *SPAST* | c.*51A>G | 3 UTR | rs6730121 |  |
|  |  |  |  |  |

* *SPAST* c.992A>T (p.I328L) was found in one patient but in none of the healthy controls. However, it was predicted not to affect the protein function, and we thus considered this a variant with unknown effect.

** *ATL1* c.766C>A (p.His256Asp) was found in a patient and none of the controls. However, at least two relatives were healthy carriers of this change, that could thus be classified as of uncertain pathogenic effect.

**Legend to supplementary figures**

**Figure S1.** Examples of families with *SPAST* and *ATL1* mutations, or changes with uncertain effect (*ATL1* p.His256Asp). Filled symbols indicated affected individuals, and asterisks subjects who were available for the genetic analysis.
