# Supplementary figures and images for: Mutational spectrum of the SPG4 (SPAST) and SPG3A (ATL1) genes in Spanish patients with hereditary spastic paraplegia
Source: BMC Neurol. 2010 Oct 8;10:89. doi: 10.1186/1471-2377-10-89 (PMC2964648; doi:10.1186/1471-2377-10-89)

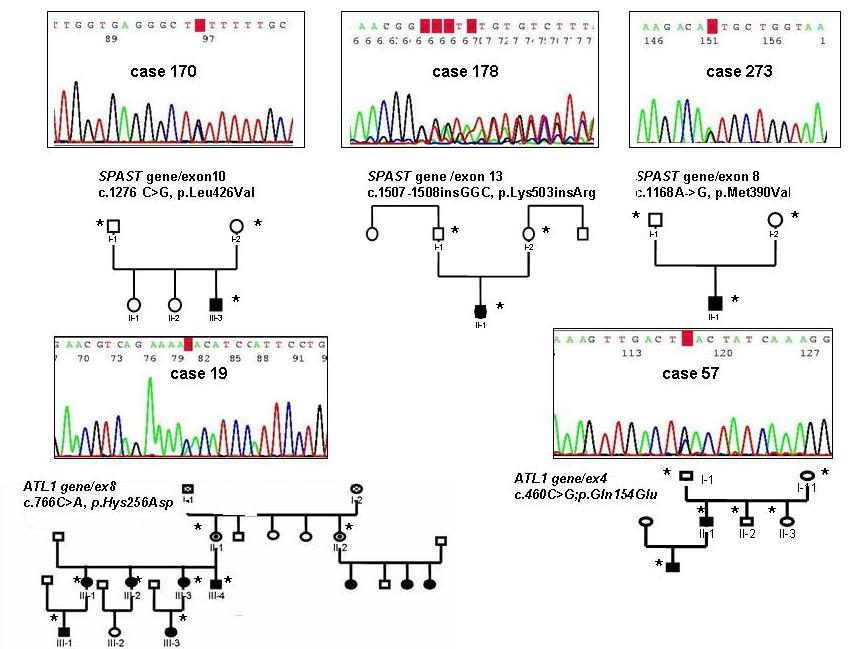

Supplement: Additional file 2 — supplementary FIGURE1 for Alvarez et al. This file contains a figure showing several examples of mutations detected. [file 1471-2377-10-89-S2.JPEG]
